# Identification of a Key Residue for Oligomerisation and Pore-Formation of *Clostridium perfringens* NetB

**DOI:** 10.3390/toxins6031049

**Published:** 2014-03-12

**Authors:** Sérgio P. Fernandes da Costa, Christos G. Savva, Monika Bokori-Brown, Claire E. Naylor, David S. Moss, Ajit K. Basak, Richard W. Titball

**Affiliations:** 1 College of Life and Environmental Sciences, University of Exeter, Stocker Road, Exeter EX4 4QD, UK; E-Mails: s.p.fernandes-da-costa@exeter.ac.uk (S.P.F.C.); m.bokori-brown@exeter.ac.uk (M.B.-B.); 2 Department of Biological Sciences, School of Crystallography, Institute of Structural and Molecular Biology, Birkbeck College, Malet Street, London WC1E 7HX, UK; E-Mails: c.savva@mail.cryst.bbk.ac.uk (C.G.S.); c.naylor@mail.cryst.bbk.ac.uk (C.E.N.); d.moss@bbk.ac.uk (D.S.M.); a.basak@mail.cryst.bbk.ac.uk (A.K.B.)

**Keywords:** NetB, pore-forming toxin, *Clostridium perfringens*, necrotic enteritis

## Abstract

Necrotic enteritis toxin B (NetB) is a β-pore-forming toxin produced by *Clostridium perfringens* and has been identified as a key virulence factor in the pathogenesis of avian necrotic enteritis, a disease causing significant economic damage to the poultry industry worldwide. In this study, site-directed mutagenesis was used to identify amino acids that play a role in NetB oligomerisation and pore-formation. NetB K41H showed significantly reduced toxicity towards LMH cells and human red blood cells relative to wild type toxin. NetB K41H was unable to oligomerise and form pores in liposomes. These findings suggest that NetB K41H could be developed as a genetic toxoid vaccine to protect against necrotic enteritis.

## 1. Introduction

Necrotic enteritis toxin B (NetB) is a recently identified β-pore-forming toxin (β-PFT) produced by *Clostridium perfringens* [1,2]. This toxin has been identified as a major virulence factor in avian necrotic enteritis (NE). According to an estimation in the year 2000, NE is causing an economic damage of around 2 billion US dollars a year to the poultry industry worldwide [3].

In a previous study, the crystal structure of the NetB heptamer was solved and showed high similarity to the structure of the heptamer formed by *Staphylococcus aureus* alpha-hemolysin (αHL) [4], the prototypic member of the related αHL-like β-PFTs family. Proteins belonging to this family are organised into three main domains according to their structure and function: rim, stem and β-sandwich. The rim domain is essential in mediating binding of the toxin to the target cell membrane, the stem domain consists of the characteristic β-hairpin involved in the penetration of the toxin into the membrane and formation of the transmembrane β-barrel structure. The β-sandwich domain, rich in β-strands, forms the protein backbone with key functions in toxin oligomerisation [5,6,7].

In general, pore-formation by αHL-like β-PFTs is suggested to occur in four-steps: (i) secretion of a water-soluble monomeric toxin by the bacterium; (ii) binding of the monomer to the membrane; (iii) assembly of the membrane-bound monomers into a multimer; and (iv) formation of a membrane-spanning β-barrel pore-structure [8,9,10]. Extensive mutagenesis studies with αHL have led to the identification of several amino acid residues important for toxin oligomerisation and pore-formation [11,12]. A pre-pore stage, in which the αHL heptamer is fully assembled, but does not penetrate the membrane, has also been well defined [8,13,14]. Like αHL, NetB has been suggested to oligomerise into a heptameric structure on the target cell surface upon pore formation [1,4]. However, the assembly mechanism is still not well understood. This study was conducted to gain information on the functional roles of selected amino acids in NetB oligomerisation and pore-formation.

## 2. Results and Discussion

### 2.1. Purification of Wild Type NetB and Variants of NetB

For site-directed mutagenesis, we have not only selected amino acids which are conserved across αHL-like β-PFTs (K41, P155 and D156) but also amino acids which, on the basis of the heptameric NetB crystal structure, we believe play critical roles in toxicity but are not conserved across αHL-like β-PFTs (K71 and D250). The latter residues were also chosen based on their proximity to residues found to be functionally important in *S. aureus* αHL. Mutation of αHL R66 or αHL D254 has been shown to reduce binding and hemolysis of rabbit red blood cells (rRBCs) [12]. Variant forms of NetB were made as described in the experimental section by site-directed mutagenesis and verified by sequencing.

**Figure 1:**
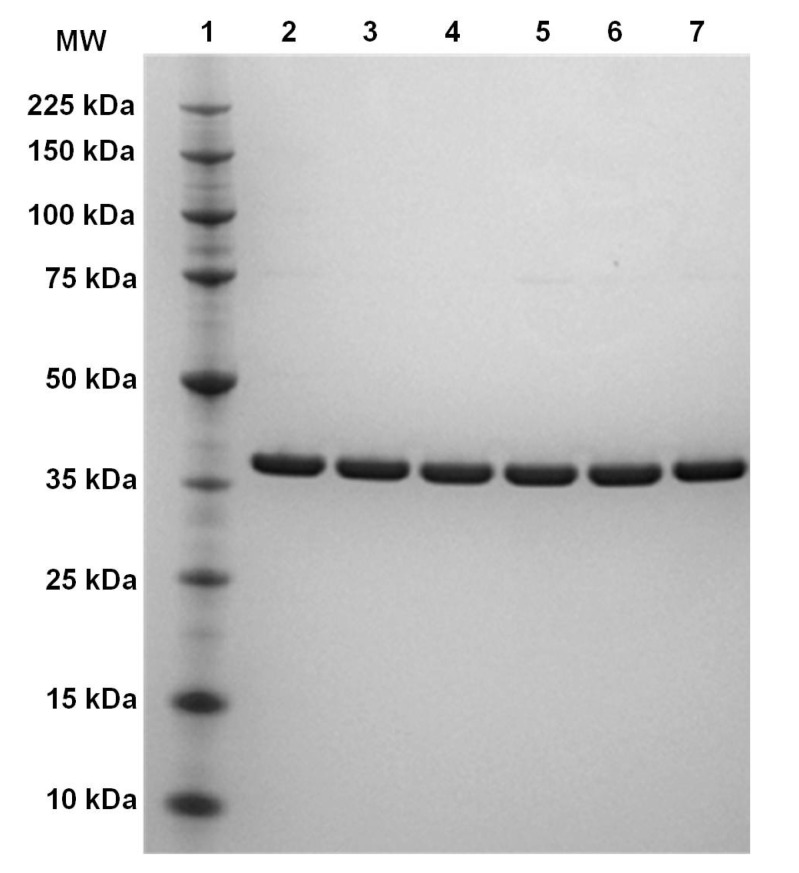
SDS-PAGE analyses of wild type NetB and variants of NetB. After protein purification, wild type NetB and NetB variants were analysed by SDS-PAGE. Lane 1: protein marker (molecular mass is indicated in kDa to the left); lane 2: wild type NetB; lane 3: NetB K41H; lane 4: NetB K71A; lane 5: NetB P155A; lane 6: NetB D156C; lane 7: NetB D250A.

**Figure 2:**
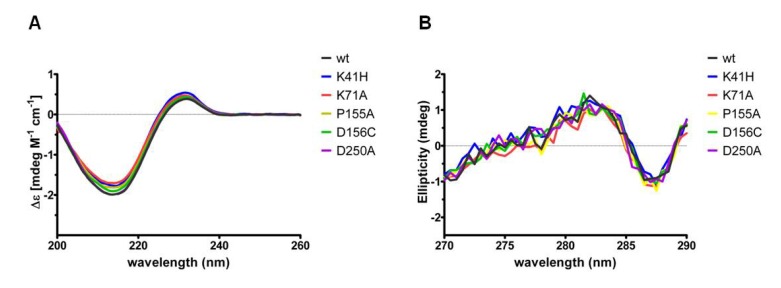
Far-UV and near-UV CD spectra of wild type NetB and variants of NetB.

Expression and purification of the variant forms of NetB resulted in similar protein yields to wild type toxin. The purified proteins were analysed by SDS-PAGE and showed a single band with an apparent molecular weight of 38 kDa (Figure 1). SDS-PAGE of purified proteins were performed three times and always showed the same protein migration pattern. Far-UV and near-UV circular dichroism (CD) spectroscopy analyses revealed very similar spectra and secondary structure contents of the NetB variants relative to wild type NetB (Figure 2; Table 1), indicating that NetB variants were folded correctly.

### 2.2. Binding and Toxicity of NetB to LMH Cells and hRBCs

**Figure 3:**
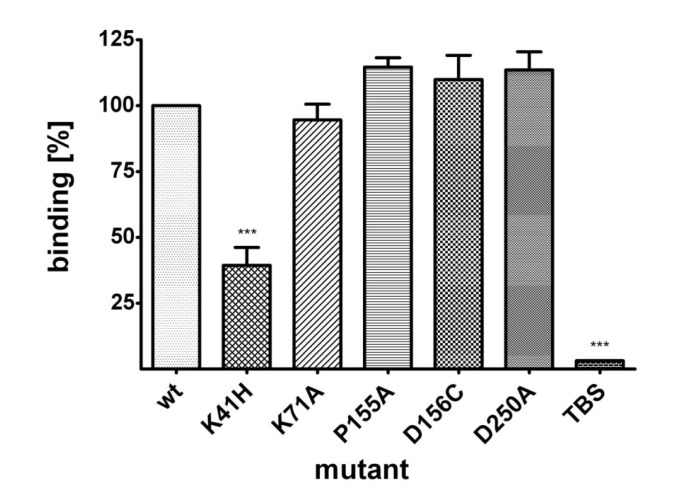
Binding of wild type NetB and variants of NetB to LMH cells. LMH cells were grown on 96-well plates and incubated with wild type NetB or variants of NetB (23 μM) for 10 min at 37°C. The degree of binding is shown relative to wild type toxin (100%). Graph represents data from four replicates in three independent experiments (data are means ± SEM; *n* = 3). Asterisks indicate a statistically significant difference (***: *p* < 0.001; 1-way ANOVA) relative to wild type NetB.

Wild type NetB and NetB variants K41H, K71A, P155A, D156C and D250A were tested for their ability to bind to the chicken hepatocellular carcinoma epithelial cell line (LMH). Proteins were incubated with LMH cells for 10 min at 37 °C and bound protein measured by an On-cell Western assay. Binding data revealed that NetB K41H was significantly impaired in binding to LMH cells, showing a reduction of 61% relative to wild type toxin (Figure 3).

**Table 1:** Protein secondary structure contents of wild type NetB and variants of NetB. Protein secondary structure contents were estimated based upon the principle component regression method embedded within the Grams 32AI/PLS software.

Protein	α-helix (%)	β-sheet (%)	other (%)
NetB wt	9.5	32.2	58.3
NetB K41H	7.2	33.2	59.6
NetB K71A	7.2	33.2	59.6
NetB P155A	8.6	32.5	58.9
NetB D156C	8.7	32.6	58.7
NetB D250A	9.5	32.2	58.3

**Figure 4:**
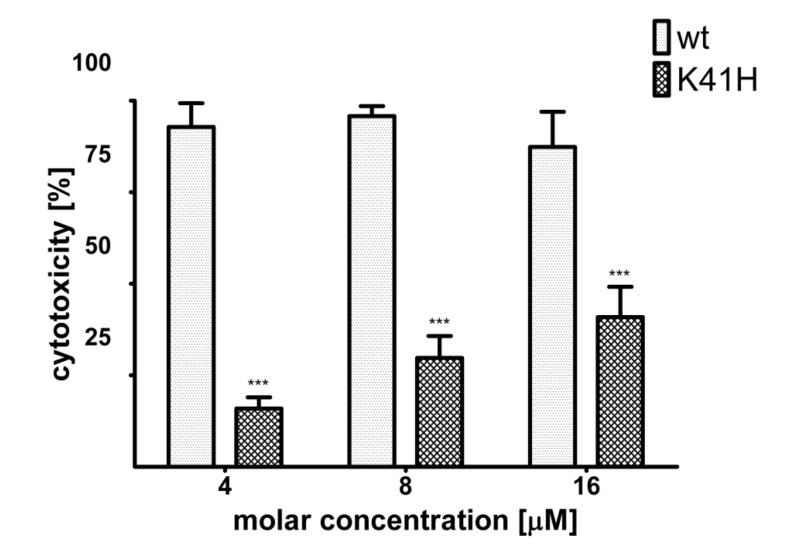
Comparison of the cytotoxic effect of wild type NetB and NetB K41H towards LMH cells. LMH cells were grown on 96-well plates and incubated with wild type NetB or NetB K41H at a concentration of 4 µM, 8 µM or 16 µM for 2 h at 37°C. Cytotoxicity is shown relative to the signal of untreated (0%) and lysed cells (100%). Graph represents data from three replicates in three independent experiments (data are means ± SEM; *n* = 3). Asterisks indicate a statistically significant difference (***: *p* < 0.001; 2-way ANOVA) relative to wild type toxin.

Next, NetB K41H was tested for cytotoxicity towards LMH cells. In a previous study, the dose of wild type NetB required to lyse 50% of LMH cells (CT_50_) was determined as 800 nM [4]. In this study, LMH cells were incubated with wild type NetB or NetB K41H at molar concentrations of 4 µM (4 × CT_50_), 8 µM (10 × CT_50_) or 16 µM (20 × CT_50_). A statistically significant reduction in cytotoxicity between NetB K41H relative to wild type NetB was shown at all toxin concentrations tested and even at the maximum molar concentration tested of 16 µM only 41% of the cells were lysed by NetB K41H (Figure 4).

**Figure 5:**
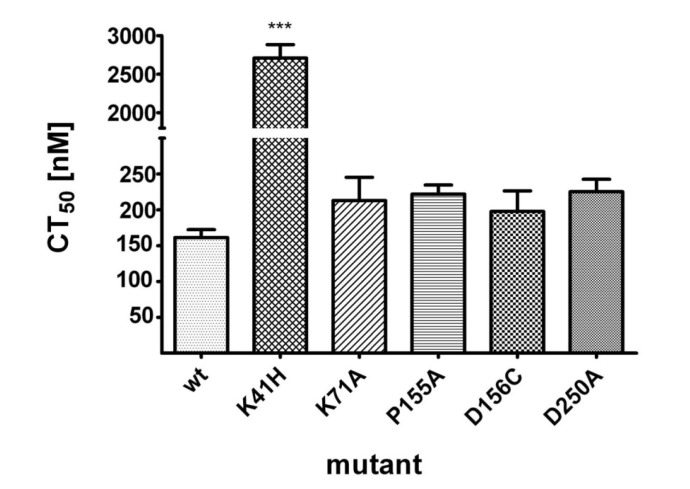
Hemolysis of hRBCs by wild type NetB and variants of NetB. Two-fold dilution series of wild type NetB or variants of NetB were incubated with hRBCs at molar concentrations ranging from 20 nM to 10 μM. The required dose to lyse 50% of the cells (CT_50_) was determined after incubation of NetB for 1 h at 37°C. Graph represents data from three independent experiments (data are means ± SEM; *n* = 3). Asterisks indicate a statistically significant difference (***: *p* < 0.001; 1-way ANOVA) relative to wild type NetB.

The hemolytic activities of wild type NetB and variants of NetB were compared by determining their median hemolytic dose (CT_50_) towards human RBCs (hRBCs). While wild type NetB showed a CT_50_ of 161 nM, NetB K41H showed a 16-fold reduced cytotoxicity towards hRBCs, with a CT_50_ of 2710 nM (Figure 5). No significant differences in CT_50_ doses were observed for the other NetB variants relative to wild type toxin.

In order to test if NetB K41H was able to form functional pores, an osmotic protection assay using polyethylene glycol (PEG) of different molecular sizes was performed. While hemolysis of erythrocytes could only be partially blocked by PEG 300, 26% in the case of wild type toxin and 15% in the case of NetB K41H, hemolysis was notably blocked by PEG 1000 by 79% and 77% for wild type toxin and NetB K41H, respectively (data not shown).

### 2.3. Oligomerisation and Pore-Formation of NetB

**Figure 6:**
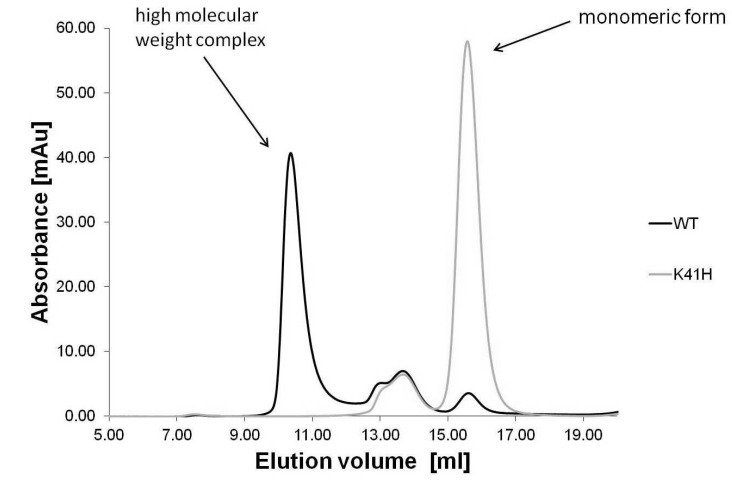
Size-exclusion chromatography of liposome-bound wild type NetB and NetB K41H. Wild type NetB and NetB K41H were mixed with liposomes, followed by detergent extraction and run on a Superose-6 size-exclusion chromatography column.

To test the ability of wild type NetB and NetB K41H to form high molecular weight complexes on the liposomal surface, detergent extracted proteins were analysed by size-exclusion chromatography. The chromatogram of NetB K41H showed an altered elution profile relative to wild type NetB (Figure 6). While most of wild type NetB ran as a high molecular weight complex, NetB K41H was mostly present as monomer.

**Figure 7:**
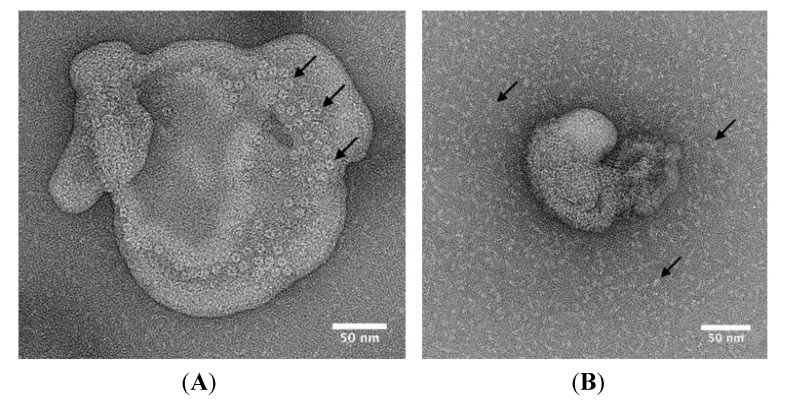
Electron microscopy of liposome-bound wild type NetB and NetB K41H. Wild type NetB and NetB K41H were mixed with liposomes and samples were negatively stained with 2% (*w*/*v*) aqueous uranyl acetate and observed using an FEI Tecnai F20 transmission electron microscope. (**A**) wild type NetB; (**B**) NetB K41H. Arrows indicate wild type NetB oligomers in panel A and NetB K41H monomers in panel B.

Electron microscopy (EM) was used to visualise NetB on the surface of liposomes. Negatively stained liposomes incubated with wild type NetB showed ring-shaped structures, which measured approximately 10 nm in diameter (Figure 7A). In contrast, no ring-shaped oligomers were observed for NetB K41H (Figure 7B).

**Figure 8:**
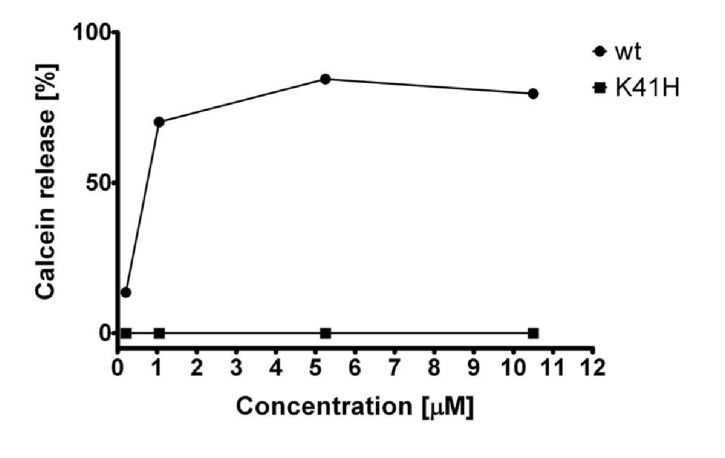
Calcein release assay of wild type NetB and NetB K41H. Calcein loaded liposomes were incubated with wild type NetB and NetB K41H at various molar concentrations and percentage of calcein release was measured after 1 h at 37°C. Graph represents data from three replicates in three independent experiments (data are means ± SEM; *n* = 3).

A calcein release assay was performed in order to test if wild type NetB and NetB K41H were able to form pores on liposomes. When wild type NetB was incubated with liposomes, calcein was released in a dose-dependent manner and over 64% of the loaded calcein was released at a toxin molar concentration of 1.05 µM (Figure 8). However, no calcein release was observed when NetB K41H was incubated with liposomes, even at the maximum molar concentration tested of 10.5 µM corresponding to a 500:1 NetB heptamer/liposome ration.

## 3. Discussions

In a previous study, we were able to show that amino acid mutations within the rim loops of NetB resulted in a significant decrease in toxicity due to reduced binding to target cells [4]. This study used site-directed mutagenesis within the β-sandwich domain of NetB to identify amino acid residues important for toxin oligomerisation and pore-formation.

NetB variants K71A, P155A, D156C and D250A did not show altered binding to LMH cells or hemolysis on hRBCs. Previous studies revealed that mutation of *S. aureus* αHL (D152C) abolished lytic activity towards rRBCs [12] and mutation of the corresponding residue in *C. perfringens* beta toxin (D167N) made the toxin unstable [15]. Surprisingly, mutation of the corresponding amino acid in NetB (D156C) did not affect activity, suggesting that this residue plays a different role in NetB.

NetB K41H showed significantly reduced binding and toxicity towards LMH cells and reduced hemolysis towards hRBCs relative to wild type toxin. Osmotic protection assays on hRBCs indicate that NetB K41H toxicity was mediated by the formation of a pore as hemolysis could be blocked by the use of PEG 1000 in a similar manner to wild type toxin. In contrast, no pore-formation was observed when NetB K41H was mixed with liposomes, possibly due to the protein-free composition or different fluidity of the liposomal bilayer. Membrane lipid composition and membrane fluidity have been shown to be an important factor for NetB oligomerisation and pore-formation [4]. It is also possible that pore-formation could have been achieved by using higher toxin concentrations. However, EM of liposome-bound NetB K41H indicate that this variant form of NetB was less efficient in binding and unable to oligomerise on the liposomal surface.

According to the solved multimeric NetB structure [4], the side chains of K41 are located in the monomer-monomer interface and thus may be involved in forming a stabilising salt bridge with the adjacent monomer. Amino acid mutation at an equivalent position in *S. aureus* γ-hemolysin (T28D) resulted in a toxin with no pore-forming activity on liposomes or human leukocytes and only slight hemolytic activity towards rRBCs [16]. Various point mutations of the equivalent amino acid position in *S. aureus* αHL (H35C, H35I, H35L, H35N, H35P, H35Q, H35R, H35S, H35T and H35W) have been shown to significantly reduce hemolytic activity towards rat and rabbit RBCs [12,17,18]. Valeva and co-workers [8,19] demonstrated that H35 is a key residue for transition of *S. aureus* αHL from the pre-pore to the transmembrane pore complex by facilitating the insertion of the stem domain into the membrane. Position K41 may play a similar role for NetB oligomerisation and pore-formation.

In a previous study, we showed that vaccination of chicken with NetB W262A was able to induce a specific immune response to NetB and significantly protect animals against NE [20]. In a similar manner, NetB K41H may have the potential to be protective when used as a genetic toxoid vaccine. A *S. aureus* αHL mutant protein in the equivalent amino acid position (H35L) has been tested in an intraperitoneal murine model and showed no lethal activity [21]. In addition, passive immunisation of mice with rabbit antiserum against αHL H35L led to significant protection against lethal challenge of wild type toxin. A recent study confirmed protection against *S. aureus* mediated pneumonia by immunisation with αHL H35L [22,23].

## 4. Experimental Section

### 4.1. Expression and Purification of Wild Type NetB

Expression of wild type NetB was carried out as described previously in *E. coli* TOP10 cells [4]. In summary, *E. coli* cells carrying the pBAD-NetB expression vector were grown in terrific broth (TB) supplemented with ampicillin (100 μg/mL) at 37 °C and shaken at 300 rpm. For protein expression, cultures were induced for 6 h by adding arabinose to a final concentration of 0.02% (*w*/*v*) at an optical density (OD_595nm_) of 0.5. Bacterial cells were harvested by centrifugation, lysed enzymatically using BugBuster (EMD Millipore, Billerica, MA, USA) and NetB was purified by Ni-NTA chromatography columns (GE Healthcare, Little Chalfont, UK) according to manufacturer’s instructions. Buffer was exchanged by size-exclusion chromatography using PD-10 desalting columns (GE Healthcare, Little Chalfont, UK) equilibrated with Tris-buffered saline (TBS; 20 mM Tris pH 7.5, 150 mM NaCl) and protein concentrations were measured with a UV-Vis spectrophotometer (Thermo Scientific, Cramlington, UK).

### 4.2. Design and Purification of Variant Forms of NetB

The following variant forms of NetB were made by site-directed mutagenesis: K41H, K71A, P155A, D156C and D250A. To keep any possible structural changes to a minimum, NetB K41 was replaced by histidine (the equivalent amino acid in *S. aureus* αHL) and NetB D156 by cysteine as replacement of the equivalent position in *S. aureus* αHL (D152) by cysteine resulted in a soluble protein [12]. None-previously mutated positions in related αHL like PFTs (K71, P155 and D250) were replaced by alanine. All amino acid numbering corresponds to prototoxin with the 30 amino acid N-terminal signal peptide sequence removed (UniProt accession number: A8ULG6). Mutants were made with the QuikChange II site-directed mutagenesis Kit (Stratagene, Santa Clara, CA, USA) and verified by DNA sequencing (Source BioScience, Cambridge, UK). NetB variants were expressed and purified as described above for wild type NetB.

### 4.3. CD Analyses

Protein integrity was assessed by far-UV and near-UV CD measurements at the Biomolecular Spectroscopy Centre, King’s College London, UK. CD Spectra were acquired on the Chirascan Plus spectrometer (Applied Photophysics, Leatherhead, UK) using either 10 mm or 0.5 mm rectangular cells in the regions of 270–290 nm or 200–260 nm, respectively. The following parameters were set: 1 nm spectral bandwidth, 0.5 nm step-size and 1.5 s instrument time-per-point. Throughout the experiment, the instrument was flushed continuously with pure evaporated nitrogen. All CD spectra were performed at room temperature and buffer baseline corrected. The far-UV CD spectra were normalised against protein concentration and path length and expressed as molar CD (Δε). For better presentation, far-UV CD spectra were smoothed with a window factor of 4, using the Savitzky-Golay method [24]. Protein secondary structure contents were estimated based upon the principle component regression method embedded within the Grams 32AI/PLS software (Adept Scientific, Letchworth Garden City, UK).

### 4.4. SDS-PAGE Analyses

Protein purity of wild type NetB and variants of NetB was analysed by SDS-PAGE on precast 4%–12% acrylamide-bisacrylamide gels (Invitrogen, Paisley, UK). All samples were heated prior to loading at 70 °C for 10 min in NuPAGE LDS sample buffer (Invitrogen). Each gel lane was loaded with 1 µg of protein. Gels were run in MES running buffer (Invitrogen) for 45 min at 200 V and stained with SimplyBlue (Invitrogen).

### 4.5. On-Cell Western™ Assay

The ability of wild type NetB and variants of NetB to bind to the chicken hepatocellular carcinoma epithelial cell line (LMH; ATTC: CRL-2117) was tested by an On-cell Western™ assay using the Odyssey CLx infrared imaging system (Li-Cor, Lincoln, USA). LMH cells were grown in Waymouth’s MB 752/1 medium (Invitrogen) supplemented with 10% fetal calf serum at 37 °C in a 5% CO_2_ incubator to 70%–80% confluency on 96-well plates and fixed with 4% formaldehyde for 20 min at room temperature. After washing the cells three times with TBS, cells were incubated with NetB at a concentration of 23 μM for 10 min at 37 °C in a 5% CO_2_ incubator. Cells were then washed three times with TBS and blocked with Odyssey blocking buffer (Li-Cor) for 1.5 h at room temperature. Cells were then incubated with an anti-Xpress antibody (Invitrogen; 1:1000) overnight at 4 °C. After washing three times with TBS, cells were incubated with IRdye 800CW goat anti-mouse (Li-Cor; 1:800) secondary antibody for 1 h at room temperature. Cells were washed three times with TBS and fluorescence was measured by the Odyssey CLx infrared scanner (Li-Cor).

### 4.6. Cytotoxic Effect of NetB on LMH Cells

The cytotoxic activity of wild type NetB or NetB K41H towards LMH cells was tested using the CytoTox96 kit (Promega Hemel Hempstead, UK). LMH cells were grown on 96-well plates as described above and incubated with 4 µM, 8 µM or 16 μM of NetB for 2 h at 37 °C. Control cells were incubated with Waymouth’s medium to determine the base line (0% lysis) or cells were freeze-thawed to determine total cell lysis (100% lysis). Cytotoxicity is shown as percentage relative to the controls.

### 4.7. Hemolysis Assay

Hemolysis assays with wild type NetB and variants of NetB were performed using human red blood cells (hRBCs) as described previously [4]. In summary, two-fold dilution series of NetB in TBS were made in a 96-well microtiter plate and hRBCs (≈4.6 × 10^6^ cells/mL) were then added to each well leading to molar concentrations of NetB ranging from 20 nM to 5 μM. Hemolysis was determined by measuring absorption at 595 nm after 1 h at room temperature with the iMark microplate reader (Bio-Rad Hemel Hempstead, UK) and using the Microplate Manager 6.0 software (Bio-Rad). The controls consisted of TBS as the negative control for 0% hemolysis and 2% (*v*/*v*) Triton X-100 as the positive control for 100% hemolysis. The median hemolytic dose (CT_50_) for causing 50% hemolysis was calculated by using the GraphPad Prism software 5.01 (GraphPad Software, La Jolla, CA, USA).

### 4.8. Osmotic Protection Assay

Osmotic protection assays with wild type NetB and NetB K41H were performed using polyethylene glycol (PEG) of different molecular sizes. Therefore, hRBCs were mixed with PEG 300 or PEG 1000 at a final concentration of 30 mM and incubated with the respective CT_50_ concentration of wild type NetB or NetB K41H. Percentage osmotic protection was calculated based on the cytotoxicity of wild type NetB (0%) in the absence of PEG.

### 4.9. Binding and Oligomerisation of NetB to Liposomes

Lipids (Avanti polar lipids, Alabaster, AL, USA) were prepared by mixing dioleoylphosphotidylcholine (DOPC), egg phosphatidylglycerol (Egg PG) and cholesterol at a ratio of 4:1:5 as described previously [4]. To allow binding and oligomerisation, wild type NetB and NetB K41H were mixed with liposomes at a protein to lipid weight ratio of 1:3 and incubated at 37 °C for 1 h. NetB was extracted from liposomes in TBS buffer containing 40 mM hexaethylene glycol monodecyl ether (C10E6) at 4 °C overnight. Unsolubilised material was removed by centrifugation at 16,000 g for 30 min at 7 °C. Detergent-solubilised NetB was analysed by SDS-PAGE and run on a 20 mL Superose 6 size-exclusion chromatography column (GE Healthcare).

### 4.10. Electron Microscopy

Wild type NetB or NetB K41H was mixed with liposomes as described above and analysed by electron microscopy (EM). Samples were prepared for EM by applying 5 µL of sample onto carbon-coated grids that had been previously glow-discharged and incubated for 60 s. Excess liquid was blotted off and grids were stained by addition of 5 µL of 2% (*w*/*v*) aqueous uranyl acetate. Samples were observed on a Tecnai F20 EM (FEI, Hillsboro, OR, USA) operating at an accelerating voltage of 200 kV.

### 4.11. Calcein Release Assay

To measure NetB pore-formation, liposomes were filled with calcein as described previously [4] and incubated with increasing molar concentrations of wild type NetB and NetB K41H (0.21 µM, 1.05 µM, 5.25 µM and 10.5 µM) at 37 °C for 1 h. The amount of released calcein was measured using a FluoroMax-3 fluorometer (Jobin Yvon Horiba, Edison, NJ, USA) with an excitation and emission wavelength of 495 nm and 515 nm, respectively. Calcein release (%) was calculated as follows:

Calcein release = (F_final_ − F_0_)/(F_max_ − F_0_) × 100 (1)

F_final_ is the fluorescence measured after incubation with the toxin, F_0_ is the background fluorescence in the absence of toxin and F_max_ is the fluorescence after addition of 1% (*v*/*v*) of Triton X-100.

## 5. Conclusions

This study showed that K41 is a key amino acid residue for NetB oligomerisation and pore-formation. As there is no NetB-based vaccine available so far, NetB K41H may have the potential to be protective when used as a genetic toxoid vaccine against NE.
